# Development of a Multi-Agency Referral Pathway for Tic Disorders in Young People in North Staffordshire

**DOI:** 10.1192/bjo.2026.11460

**Published:** 2026-06-30

**Authors:** Bindu Poornamodan, James Boardman, Philippa Marshall, Caroline Groves, Waheed Abbasi

**Affiliations:** 1 North Staffs Combined Health Care NHS Trust, Stoke on Trent, United Kingdom; 2 University Hospital of North Midlands, Stoke on Trent, United Kingdom; 3 Manchester University NHS Foundation Trust, Manchester, United Kingdom; 4 Staffs Stoke ICB, Stoke on Trent, United Kingdom

## Abstract

**Aims:**

Tic disorders are the most common movement disorder of childhood with a lifetime prevalence rate of 5% for transient tic disorders and 0.7% for Tourette syndrome. 20% of school-aged children may be affected by tics at some point. Most tic disorders are transient and do not require treatment. Chronic tic disorders if causing distress to the young person would require treatment.

Management of tic disorders requires a multidisciplinary approach with paediatricians, psychologists and psychiatrists working together depending on comorbidities. General practitioners are often unclear about the most appropriate service to refer young people to, causing delay in young people accessing the right support. This led to discussions about creating a pathway for children and adolescents with tic disorder in the community setting.

Aims were to create a referral pathway for children and young people with tic disorders in North Staffordshire; to provide a seamless service for young people, by developing a referral system with clear guidance to genera practitioners so that young people can access the right support in a timely fashion.

**Methods:**

A working group was formed with Paediatricians (3 and 4) from University Hospital of North Midlands and a Psychiatrist (1) and Psychologist (2) from the CAMHS services. A general practitioner (5) who represented ICB was also part of the working group.

The group met regularly in 2023 and created a pathway which enabled the general practitioners to refer to the most appropriate team. A referral form was also created to guide the referring clinician. This was presented to a group of general practitioners and amendments were made accordingly. The pathway was approved by the ICB and implemented in March 2024.

**Results:**

Introduction of the tic disorder pathway helped primary care colleagues to make referrals to the right team and helped reduce waiting times.

**Conclusion:**

Creation of this pathway helped primary care clinicians to assess and refer young people without referrals being redirected to different teams. This has reduced the time from referral to treatment for young people with movement disorders. The development of the pathway has positively impacted on working relationships between primary and secondary care colleagues.

